# Reviews of Books

**Published:** 1921-11

**Authors:** 


### REVIEWS OF BOOKS.

**TECHNIQUE OF THE TEAT AND CAPILLARY GLASS TUBE.** By SIR A. E. WRIGHT and LEONARD COLEBROOK. (London: Constable & Co. Price 42s.)

To physician and to surgeon alike we would at once say, "neglect the title and get the book." The title is in keeping with the intensely practical mind of the author. It is a text around which Sir Almroth builds the Science of Healing. The Technique of the Teat and Capillary Glass Tube is a description symbolic of the Science of Industrialism. From the working table, the glass tubing, the cutter, and the rubber teat, medicine and surgery are attacked in their empirical foundations throughout a climax of method, precision, ratiocination, and result, which are a striking demonstration of modern research.

Every laboratory worker knows the value, in economy and precision, of the endless finesse in technique, which is continuous during operation, but, except to his immediate colleagues, little other than result is transmitted. From this book, the sum of the work of Sir Almroth Wright and many colleagues, the beginner in laboratory work, no matter how gifted in idea, will have a jumping-off ground which may save a lifetime of plodding, and facilitate the achievement of further success in the ultimates of biochemistry.

Chapters on apparatus, glassworking, and pipette construction lead on to the principles of the technique. In natural sequence the methods of obtaining blood and preparing blood-films follow, and soon we are involved in the fascinating intricacies of blood-testing. In the appendices to Chapter VII physician and surgeon will find much of interest and of clinical value concerning the treatment of thrombosis and the arrest of hæmorrhage, and will receive enlightenment on the causation and treatment of shock and gas-gangrene, and on the significance of the anti-tryptic function of the blood.

The measurement of the agglutinating, bactericidal, and bacteriolytic power of the serum are carefully treated in a chapter which will appeal to the regular laboratory worker. The technique for measuring the opsonic power of the blood is described in a manner which will readily impress physician and surgeon to whom its clinical value is considerable.

The further study of the biological functions of leucocytes leads on to Sir Almroth's wonderful work on the study of wounds and of surgical healing. With his findings no surgeon can disagree, but from the arguments dictating practice there may be serious difference of opinion. The knowledge of the processes in infected wounds should guide every surgeon to sound "first principles" in his practice; for the rest it must be said of success in surgery as in all else that "the man counts." The logical sequence of practice based on Sir Almroth's work may not frequently, if at all, be attainable, but the facts are there to guide the knowing hand to success by available method.

A work by Sir Almroth Wright would not be complete without evidence of the polemical insistence of his outlook. In the preface we have it announced, as part aim of this volume, that "it is to bring it home to the general body of the medical profession that clinical experience—or shall we say observation?—unaided by apparatus and technique is, for the purposes of research, of infinitely little account." Probably none will gainsay this. But the author goes on to deal somewhat bitterly and unrecognisingly with Empiricism, to which perhaps he owes his scientific existence; and, an intensely practical man himself, gibes at "your plain, practical man" in a hackneyed conventional way. In Chapter IX every scientific worker will applaud his consideration of the "code of morality which ought to come into application" in operations, but may be little inclined to subscribe *in toto* to the somewhat drastic eschewing of the "mathematical statistician" in biological study, which is displayed in an appendix to this chapter.

This book is a sound production; figures and plates are accurately and elegantly depicted; and errors are very rare, though one or two lapses indicate that we cannot yet say with Denys, "*le diable est mort.*"—A. M.

**THE PRINCIPLES OF PREVENTIVE MEDICINE.** By R. TANNER HEWLETT, M.D., F.R.C.P., D.P.H., and A. T. NANKIVELL, M.D., D.P.H. (J. & A Churchill, Great Marlborough Street, London. Price 21s. net.)

AFTER reading this book we are wondering if the dry-as-dust text-books on public health really were a necessary evil. There are so many of these, and on the whole they are so dull, that we welcome this experiment by Professor Hewlett and Dr. Nankivell, who have produced a book on "The Principles of Preventive Medicine," which is not only readable, but occasionally entertaining. Some of the book, indeed, can be read a second time over, which is a severe test, even for a public-health novel.

The book was written for medical students, and we hope not only that they will read it, but also that they will be examined on the subject-matter that it and other public-health books contain. The authors, however, have attempted the impossible, for it is not reasonable to believe that the whole science of preventive medicine can be condensed into a book of 536 pages, and necessarily some of their chapters are sketchy. But, despite the impossibility of their task, they have succeeded in crowding their book with interesting and valuable material, which might well be instructive, not only to students, but to all medical men and sociologists.

They seem to have a taste for the nicely turned phrase, of which we cannot refrain from quoting a few examples: "It is certainly better, if one becomes a collector, to collect stamps rather than gonococci." "Leaders of men, even Napoleon, cannot win battles when they are fighting with chronic indigestion." "The word sanitation to the popular mind conveys the impression of well-

**Reviews of Books - (*cont.*).**

ordered drains, and they picture the sanitary department as a home of corporation officials who are nothing more than dignified and overpaid plumbers." "Snobbery and respectability are not vices of which the wealthy classes have the monopoly." "The windows (of the slum house) are never opened, and the house at the end of the week still retains the smell of last Sunday's dinner, with a *mélange* of beer, tobacco, humanity, and mice." To what extent this quality of writing is justified in a scientific text-book we must leave each of its readers to determine for himself, although personally we consider that in this instance the end justifies the means.

That Professor Hewlett and Dr. Nankivell owe much to the writings of Sir George Newman is obvious from their many quotations from his works and from their tribute to him in their preface. They owe much also to English literature, which they quote frequently to advantage. Their quotations range from the Book of Job to "The Young Visiters," taking in, *en passant*, the Church Catechism, Shakespeare, Ronsard, Borrow, Wells, Shaw, and a score of other writers. They refer on page 126 to Evan Harrington, whose mother knew that he would not go philandering when he was safe across a horse. As our authors are readers of George Meredith, we would ask them to consider Mr. Goren, the master tailor in "Evan Harrington." Mr. Goren loved his craft; he believed that he had not succeeded the millions of antecedent tailors in vain, for he had made a great discovery—namely, that of designing breeches which had a correct Balance. Mr. Goren's Balance was an invention of which he was proud to boast. There are balances and balances, and our authors seem to lack one of the varieties. There are several undisputed sociological evils which are considered in "The Principles of Preventive Medicine" and discussed, it may be, with a lack of balance. It is not necessary to overstate a good case, to protest too much against an evil. Teachers must, of course, be dogmatic, and teach that twice two are certainly, and not probably, four; but the authors of this book are inclined too much to insistence and overstatement. Perhaps in their next edition they will consider the peace that reigned in Mr. Goren's shop, the result of his having discovered a true balance. —E. D.

**MATERIA MEDICA AND THERAPEUTICS.** By J. Mitchell Bruce and Walter J. Dilling. 12th [illegible] edition. (Cassell & Co.)

Since "Mitchell Bruce" was first published thirty-seven years ago twelve revised editions and many reprints have appeared. A book with such a record obviously fulfils its purpose.

Previous editions have established a reputation for usefulness, which will be enhanced by the new edition. The form of the book remains the same, but the authors have found room for a good deal of new matter. They include, for instance, short but useful accounts of mineral-water spas and invalid diet—two subjects which are usually crowded out of the student's curriculum, but are of great importance to the practitioner, who will find the information thus obtainable in a condensed and handy form very useful to him.

The first and larger portions of the book give the essential facts concerning all the drugs of the British Pharmacopœia, official and non-official preparations of proved value and the method of their prescription and administration. The newer drugs, such as benzyl, benzoate, and ethanesal, receive adequate notice and the various modern preparations of arsenic are described. Colloidal preparations, vaccines, and vitamines are briefly but adequately discussed.

The dosage of all drugs is given in imperial and metric measures, and clear instructions are given for prescribing according to the latter system.

For the student materia medica is almost entirely a matter of memorising strengths and doses, but this book, while containing all that is necessary to ensure the smiling approbation of an examiner in these directions, also demonstrates how much more there is in therapeutics than the administration of an elegant mixture. Electrical treatment in all its varieties, ionisation, massage, exercises, and radiotherapy are described and the indications for their use pointed out without exaggeration of their value.

The form of the book is convenient and the type clear. There is small scope for criticism, but it is unfortunate that the correct and emphatic statement on page 281 that digitalis does not raise the blood-pressure in man should be followed by an apparent contradiction on page 540. In the necessarily brief account of cardiac disorders no mention is made of flutter, and the remedial value of adrenalin in heart-block is omitted.

The student will discover in this edition, as his predecessors have found in past editions, that the dry bones of drugs and doses can be made to live; and the practitioner will find a handy and reliable manual of reference which will be invaluable to him.—C. E. S.

**BIOLOGICAL CHEMISTRY.** By H. E. Roaf, M.D., D.Sc. With 47 Diagrams. Pp. xvi, 216. (London: Methuen and Co., Ltd. 1921. Price 10s. 6d. net.)

The chemical processes that take place within the living cell, whether of the animal or vegetable type, are singularly complex, and require for their elucidation a knowledge of many different branches of chemical science. Professor Roaf has given in a small compass an excellent exposition of the main facts of organic chemistry and their practical bearing upon modern biological problems. After dealing with the laws of chemical reaction in so far as they are concerned with certain biological processes, the second section of the book treats of the accumulation of available energy by the absorption of the radiant energy from the sun by the green plant. This is followed by an account of the catabolic processes that characterise the vital activities of the body, in which much recent work is incorporated. In this section is a description of the deficiency diseases, with some telling illustrations.—G. N. M.

**Reviews of Books—(*cont.*).**

**TREATISE ON FRACTURES IN GENERAL INDUSTRIAL AND MILITARY PRACTICE.** By JOHN B. ROBERTS, A.M., M.D., F.A.C.S., and JAMES A. KELLY, A.M., M.D. Second Edition. Revised and entirely reset. Pp. viii+755. With 1081 illustrations. 1921. (Philadelphia and London: J. B. Lippincott Company. Price 42s. net.)

Six years have elapsed since the publication of the first edition of this book, and many advances have taken place in that time. The clinical opportunities afforded by the Great European War have taught us much in regard to the treatment of fractures, and have modified many of our previous conceptions. Another agency which has forced a revision of our older methods is the payment by industrial firms for the hospital care and treatment of injured employees.

The present edition has been revised, completely reset, and greatly added to. It consists of over 750 pages, with 1,081 illustrations, consisting of radiograms, photographs, and diagrams. The book consists of thirty-one chapters; the first five deal with general considerations, the next twenty-four deal with the fractures in detail, and the remaining two deal with obstetric fractures and industrial and war fractures.

In the preface the authors state twenty-six very valuable propositions, which contain the essence of the teaching contained in the volume. The last proposition states that no special form of splint or apparatus may be substituted with safety for the requisite knowledge on anatomy, pathology, and mechanics, and with this in view the opening part of each chapter deals briefly with the anatomy and the surface markings of the parts; then come statistics and after results, and in many cases a brief summary of the treatment is given again at the end of the chapter.

The chapter on obstetric fractures is a singularly interesting and instructive one, although in practice they are observed infrequently, and few books on fractures deal with them.

The final chapter deals with industrial and war fractures. Many of the fractures seen in civil life are identical with those seen in war, and consequently the treatment does not differ. The treatment and reconstruction of these cases is based on an extensive experience in the recent great war, and those surgeons who have the care of employees of factories, mines, shipyards, etc., will do well to follow the treatment laid down in this treatise.

This work is complete, and has been compiled with painstaking attention to detail. The varieties of fractures are shown by well-selected radiograms, the displacement of the fragments by accurate drawings, and in many cases the methods of reduction and application of retentive apparatus are presented by photographs. It is a clear, concise, and systematic presentation of the subject of fractures, and should prove of use not only to the student and medical practitioner, but also to the surgeon of experience.—T. P. N.

**CHINA AND MODERN MEDICINE.** A Study in Medical Missionary Development. By HAROLD BALME, F.R.C.S., D.P.H., Dean of the Medical School, Shantung Christian University, Tsinan. With Preface by Sir Donald MacAlister, K.C.B., M.D. (London: United Council for Missionary Education, 2 Eaton Gate, S.W. 1921. Pp. 224. Price 5s. net.)

In tracing the growth of medical science in China from the time of Colledge to the present day, the author of this interesting study has indicated the methods by which the principles and practice of Western medicine have been presented to the native mind. The seed was sown in 1838, when Peter Parker " opened the gates of China with a lancet when European cannon could not heave a single bar." He and other medical missionaries were among the pioneers of modern medicine in every part of the vast empire.

The barriers of tradition and superstition have been gradually overcome by the weapons of kindness, self-sacrifice, and a living demonstration of the superiority of scientific medicine over the native art. Even the tragic events of 1900, when four medical men were numbered among the martyrs, proved but the prelude to an extraordinary advance in the years that followed. Of the three hundred mission hospitals in full work to-day in China, some 80 per cent. have been established during the last two decades. One of the most encouraging features of all has been the manner in which the Chinese have themselves built hospitals and dispensaries of their own. In the words of Sir Donald MacAlister, who contributes a preface, there can be no doubt that Chinese medicine, " guided into the true path by the devotion of men like Professor Balme and his colleagues, will in due course enrich the science of the world with new discoveries in China's own immense and unexplored fields." As a cure for much of the present-day pessimism and as a vivid exposition of Christian medical missions, this book may be cordially recommended.—G. N. M.

**SURGICAL WARD WORK AND NURSING.** By ALEXANDER MILES, M.D., F.R.C.S. Fourth edition. (The Scientific Press, Ltd., 28-29 Southampton Street, Strand, London. Price 10s. 6d.)

Writings from the pen of this author are sure to find a premier place among the standard works on the subject upon which he writes, and the present book will take a no less merited position than that of the three previous editions. In it Dr. Miles has revised and amplified in accordance with the strides and advances of surgical science the practical essentials and manifold details coming within the province of the modern surgical nurse. From primary conceptions to advanced developments the subject is handled in brief yet explicit phraseology, providing an entertaining as well as an instructive study, aided by a generous series of illustrations throughout. The book is to be recommended for its completeness and an absence of that overlapping into spheres which, although allied, have no direct concern with the student, to whom the book is addressed.—A. I.

